# Prospective Association Between Weight Variability and Subsequent Long‐Term Weight Loss in the CALERIE Study

**DOI:** 10.1002/oby.70235

**Published:** 2026-06-18

**Authors:** Branislav Jovanovic, Simar Singh, Fengqing Zhang, Edward A. Williams, Susan B. Roberts, Michael R. Lowe

**Affiliations:** ^1^ Department of Psychological and Brain Sciences Drexel University Philadelphia Pennsylvania USA; ^2^ Department of Psychiatry and Behavioral Sciences University of California San Francisco California USA; ^3^ The Geisel School of Medicine Dartmouth College Hanover New Hampshire USA

**Keywords:** weight change, weight fluctuation, weight loss, weight maintenance, weight variability

## Abstract

**Objective:**

Higher short‐term weight variability (WV) predicts greater subsequent weight gain in weight‐stable adults and toddlers and lower long‐term weight loss among adults with overweight or obesity who previously lost weight. We extend prior work by examining the impact of naturally occurring WV on subsequent weight loss (weight at month 3 minus later weights) in individuals without obesity enrolled in the Comprehensive Assessment of the Long‐term Effects of Reducing Intake of Energy (CALERIE) study.

**Methods:**

Participants (*N* = 143) followed a 25% caloric restriction diet over 2 years. WV was calculated as the root‐mean‐squared‐error of individuals' weights over the first 12 weeks. Multilevel models examined WV's prediction of subsequent weight loss at 6, 12, 18, and 24 months relative to month 3, adjusting for baseline BMI, 12‐week weight change, and demographic variables. Sensitivity analyses further included time‐varying adherence and eating behaviors (disinhibition, restraint, craving).

**Results:**

Higher WV was associated with less subsequent weight loss at 6 months (*B* = −0.38, 95% CI [−0.63, −0.13], *β* = −0.16, *p* = 0.003). The interaction between time and WV was not significant; analyses suggested the association extends through year 1.

**Conclusions:**

Short‐term WV predicted lower subsequent weight loss in adults without obesity. Once its biobehavioral mechanisms are understood, WV might provide insight into humans' innate capacity for long‐term weight stability and inform future mechanistic research and clinical interventions.

**Trial Registration:**

ClinicalTrials.gov registration: NCT00427193

## Study Importance

- What is already known?
  ○ Weight variability (WV) consistently predicts weight change over time across clinical and non‐clinical populations.
  ○ Among individuals with obesity who are losing weight, higher WV has been prospectively associated with less weight loss.
- What does this study add?
  ○ Higher WV during weight loss is prospectively associated with reduced subsequent weight loss in individuals without obesity or notable health problems.
  ○ Analyses suggest this association may persist through year 1, even after accounting for eating behavior and weight‐related covariates.
- How might these results change the direction of research or the focus of clinical practice?
  ○ WV may serve as an early marker for adverse weight‐related outcomes.
  ○ WV might provide insight into body regulation and be a screening tool for at‐risk individuals who might be more prone to obesity.

## 1 Introduction

Human survival depends on the regular consumption of energy. In the modern world, this pattern of consumption culminates in roughly 1 million calories consumed per year by the average adult [1]. Across people maintaining weight (roughly half of the U.S. population over a 10‐year span) [2], energy intake closely matches energy expenditure, evident by very high weight stability despite fluctuations in daily energy intake [2, 3]. Given that most humans do not routinely track daily variations in their energy intake and energy output to maintain weight, the remarkable degree of weight stability achieved over time may be attributable largely to an automatic, homeostatic process.

### 1.1 Weight Variability

Even individuals with high weight stability over long periods show small daily or weekly variations in body mass around their stable baseline level [4]. These small variations in weight—called weight variability (WV)—are not surprising, given the number of internal (e.g., neural and endocrine signals) and external (e.g., food‐abundant environments) inputs that affect energy intake and output [5], as well as measurement error and variations in fluid balance [4]. It is surprising, however, that WV associates with weight change and medical outcomes, despite being a presumably normative phenomenon. For instance, in both weight‐stable individuals [6, 7, 8] and those undergoing weight loss [9, 10, 11], both short‐term (over 3 months) and long‐term WV (over 1 year or more) predict weight change over time.

Short‐term WV is also prospectively associated with increased risk of cardiovascular events independent of BMI [12, 13]. Further, long‐term WV correlates with an increased risk for all‐cause [14], heart‐, cancer‐ or other‐cause‐related mortality [15, 16, 17], cardiovascular disease [17], type 2 diabetes [18, 19], insulin resistance [20], psoriasis [21], and metabolic syndrome [22] in both clinical and community samples.

It is intriguing that similar measures of WV have been consistently related to both future weight change and to worsening of numerous medical conditions; however, it remains unclear whether the basis for these associations is shared across the two literatures. Nevertheless, these studies suggest that short‐term WV not only reflects a stochastic process, but may also reveal systematic information that is predictive of medical outcomes and body weight trajectories. In other words, although these small, bidirectional fluctuations in body weight could be thought of as simply noise in the data, there is consistent evidence that the between‐subjects level of such variability also conveys a meaningful signal, reflecting susceptibility to greater weight gain (or, after a weight loss, to poorer weight loss maintenance) over months and even years (for a review, see et al. [3]).

### 1.2 Current Study

Because studies to date have not probed the WV effect among individuals without obesity, it remains unclear whether the prospective association between WV and weight change is a result of an individual's weight status or part of an underlying and yet unexplained process. Investigation into this area may deepen our understanding of WV and its mechanisms and help to further ascertain WV's prognostic value in clinical settings. For example, if high WV is a reliable transdiagnostic risk factor, then practitioners could anticipate and potentially address future weight gain or an unsuccessful weight loss attempt.

To answer this, we tested whether the previous WV findings generalize to a weight loss cohort consisting entirely of individuals without obesity and without any physical or mental health ailments (i.e., a healthy sample) as part of the Comprehensive Assessment of the Long‐term Effects of Reducing Intake of Energy (CALERIE) study [23]. The CALERIE study was a multisite block randomized controlled trial comparing weight loss among healthy individuals without obesity (i.e., BMI range = 22–28 kg/m^2^) who were assigned to an intensive recommended 25% caloric restriction (CR) diet or to a recommended ad libitum control diet. Importantly, participants included in the study had no history of notable medical conditions, no current psychiatric or behavioral problems, and no abnormal laboratory markers, and they were not using medications known to influence body weight. Therefore, the sample in this study is unique not only because it examined participants without obesity, but also because the sample was chosen to be “extra‐healthy.” As a result, if WV's effect on weight loss extends to this sample, we can be more confident that the effect is *not* due to weight status or an underlying medical condition.

**FIGURE 1:**
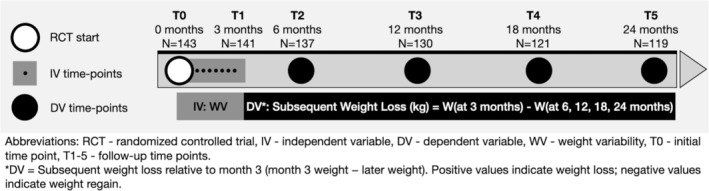
Timeline of the study from T0 to T5 highlighting prospective measurement of weight variability (WV) and 3‐month referenced weight loss over time.

Our primary aim examined the prospective association of WV on subsequent weight loss after month 3 in individuals without obesity over time (6–24 months), while controlling for relevant demographic, weight‐, eating‐, and RCT‐related covariates. We hypothesized that higher WV will be associated with less subsequent weight loss over time, as observed previously [4]. Consistent with our prior work [6], we also examined the robustness of this association while accounting for eating behaviors that might impact participants' weight change and weight fluctuations in our model (e.g., disinhibition). Figure 1 illustrates the study timeline, indicating the timing of independent and dependent variable measurements and the duration of the study.

Our secondary aim examined the interaction of time with WV to determine if an initial effect of WV on weight loss is found and whether it persists over and/or interacts with time. Prior work has only examined the strength of the effects cross‐sectionally at individual follow‐up points [9, 10], so it remains unclear if the effects of such associations change significantly over time.

## 2 Method

### 2.1 Participants

We analyzed data from the individuals recruited as part of the CALERIE study (ClinicalTrials.gov registration: NCT00427193). The CALERIE study aimed to measure markers of aging in humans randomized to an ad libitum 25% CR diet over 2 years [23]. Individuals randomized to the 25% CR diet (the analytic sample for this secondary analysis) had energy intake assessed via doubly labeled water to set a person‐specific diet achieving 25% restriction [23]. During the initial month, participants received complete CR meals, individual and group counseling, and guidance on monitoring nutrient intake to support adherence [23]. Most comprehensive evaluations relevant to weight loss and other outcomes were performed at baseline and 6‐, 12‐, 18‐, and 24‐month follow‐ups [23]. Further details about the multisite CALERIE study design have been reported elsewhere [23, 24]. All individuals consented to research participation, and the study was approved by each site's respective Institutional Review Board [24]. Additional CALERIE requirements included the absence of notable health problems, abnormal laboratory results, no recent substantial weight loss, no history of or current eating disorders, no behavioral or psychiatric problems, no engagement in heavy physical exercise (i.e., 30 min or more, five or more times per week), no regular medication use (except contraceptives), and being of age 20 to 50 for men and 20 to 47 for women [24]. A total of 143 participants were included in this secondary data analysis.

### 2.2 Weight Variability

All weights (in kg) used in the analyses of 12‐week WV and long‐term weight loss were obtained in‐clinic, by a trained CALERIE staff member. Participants were weighed in the morning following an overnight fast and after voiding [25]. Additionally, participants were asked to remove their shoes and wear a pre‐weighed hospital gown before the measurements [25]. Details about the specific equipment used, calibration and weight measurement methods, and quality control procedures are reported in the CALERIE Evaluation Protocol [25]. Consistent with prior research [6, 7, 8, 26], weight over the first 12 weeks (i.e., 3 months) was used to calculate WV. Each participant was weighed weekly during the first 4 weeks; later, they were measured every other week for a total of nine assessment points used to estimate the 12‐week WV. The ‘lmer’ function [27] in RStudio (v.2023.09.1) was used to fit multilevel models for weekly weights with increasing complexity, starting from an empty‐model and then adding higher order terms (including linear, quadratic, cubic fixed, and random effects). Participants' starting value and changes over time were incorporated into the model estimation procedure, and time was nested in respondents. Fixed and random effects were selected stepwise, retaining terms only when likelihood ratio tests showed they significantly improved model fit [28]. The final and optimal model included linear and quadratic fixed effects across participants and linear and quadratic random effects within participants to capture individual weight trajectories (see online Supporting Information for model specification and code). The root‐mean‐squared‐error of residuals around this regression line was calculated, representing the WV construct. Finally, sensitivity analyses using alternative operationalization of WV confirmed that our approach is robust and appropriate for capturing participant‐level variability (online Supporting Information).

### 2.3 Subsequent Weight Loss

Clinic‐based weights (in kg) were again obtained at each study assessment point (i.e., 3, 6, 12, 18, and 24 months). Measurements were taken in the morning with participants wearing a hospital gown and no shoes, following a 12‐h fast [25, 29]. Because we were interested in testing WV's prospective *prediction* of weight change over time, the 12‐week WV (i.e., main predictor) had to precede weight change (i.e., the outcome of interest). Consequently, weight change is defined as the difference in weight at 6, 12, 18, and 24 months post baseline relative to the weight measured at 3 months, resulting in no overlap between the WV measurement period and the weight loss period. Finally, because participants in the CR group aimed to lose weight, we defined subsequent weight loss as weight change relative to month 3, coded such that positive values indicate weight loss and negative values indicate weight regain. For example, if a participant weighed 80 kg at month 3 and 77 kg at month 6, this corresponds to +3 kg (weight loss), whereas 82 kg corresponds to −2 kg (weight regain).

### 2.4 Statistical Plan

All analyses were conducted in RStudio (v.2023.09.1). To address our first hypothesis, we conducted a general linear mixed model to test if an increase in WV was associated with a significant decrease in weight loss from 6 to 24 months. Given the nested structure where time was nested in respondents, all models included a random intercept for participants and a random slope for the assessment point (i.e., measured in months). The fixed effects included WV, linear time (months since beginning of the study, but centered at 6‐month time point), and the interaction between the WV and time.

We tested two models: Model A includes covariates used in all prior studies and sociodemographic factors. Model B was specified as a sensitivity (robustness) model that additionally included time‐varying percent CR achieved and eating behavior variables assessed during follow‐up. These variables were measured at each assessment wave and were modeled concurrently with the corresponding weight outcomes. They are theoretically and empirically linked to energy balance and weight regulation and have been incorporated in prior work when available [6]. More specifically, model A controlled for the following effects: weight change over the WV period (i.e., weight at Week 1 minus Week 12) and baseline BMI, consistent with prior research [6, 7, 8, 26]; intervention site, to control for any site‐specific effects on intervention administration as reflected in weight change [29]; age and sex, to control for their associations with metabolism [30, 31], hormonal regulation [32, 33, 34], and body composition [35, 36, 37]; and income, as a social determinant of health, to control for access to food, health care, and opportunities for physical activity. Regarding other social determinants of health, we did *not* control for race or ethnicity, given unequal distributions across subgroups, or education, given high covariance with income and potential multicollinearity issues while lowering our statistical power. Model B also controlled for the time‐varying eating behavior constructs of restraint (i.e., conscious restriction of food intake) and disinhibition (e.g., overeating in response to different stimuli) from the three‐factor eating questionnaire [38], and craving (using a normalized 28‐item food craving score) [39]. Because these variables were assessed during the follow‐up period, they may be influenced by earlier WV and could lie on the causal pathway between WV and subsequent weight change. At the same time, prior work indicates that these factors are associated with both weight change and WV [6, 40], providing justification for their inclusion in model B. Additionally, variation in percent CR achieved (i.e., a measure of adherence) may influence both WV and weight loss [41]. Accordingly, model B also included percent CR at months 6, 12, 18 and 24, derived using the energy intake values (calculated through the doubly labeled water method). Income, gender, and site were the only categorical covariates in our analyses; remaining variables were treated as continuous.

All model assumptions were met (online Supporting Information). Extreme outliers were retained as they did not affect results, and missing data due to attrition were handled using maximum likelihood estimation [28] in mixed models.

### 2.5 Power Analysis

We used a combination of G*Power [42] and calculation of design effect for the regression coefficient of a time‐invariant predictor [43] to calculate statistical power. Prior literature estimates a small effect of WV on weight loss; *ΔR* ^2^ = 0.04 at 12 months using 12‐week WV [10]. The estimate of the design effect per the methodology of Snijders [43] was 0.36. Consequently, at a significance criterion α = 0.05, effect size *ΔR* ^2^ = 0.04, we would need 70 participants to be powered at 0.80. To explore the moderation of WV and time, under the similar assumptions and the effect size *f* ^*2*^ = 0.02, we would need 145 people. We were sufficiently powered to detect the main effects and slightly underpowered for the interaction effect.

## 3 Results

### 3.1 Participant Characteristics and Correlation Matrix

The majority of participants were female (*n* = 99, 69.2%) and White (*n* = 111, 77.6%). The average age was 38.0 years (SD = 7.3). On average, 12‐week WV was 0.4 kg (SD = 0.2), and weight loss over this 12‐week period was 6.4 kg (SD = 2.2). The subsequent weight loss over time was consistently between 1.7 and 2.8 kg (see Table 1 for complete descriptives).

**TABLE 1:** Descriptive statistics of the key variables used in the analysis (*N* = 143).

Categorical variables	Frequency	Percentage (%)
Gender		
Male	44	30.77
Female	99	69.23
Ethnicity^a^		
White	111	77.62
Black	15	10.49
Other/multiracial	6	4.20
Asian	11	7.69
Education^b^		
HS graduate	2	1.40
Some college	21	14.69
Undergraduate degree	67	46.85
Graduate degree	53	37.06
Income		
$0–$39,999	17	11.89
$40,000–$79,999	45	31.47
> $80,000	81	56.64
Site		
Site 1	46	32.17
Site 2	53	37.06
Site 3	44	30.77

Continuous variables	*n*	*M*	Range	SD
Age	143	37.96	20.72–50.79	7.34
12‐week WV (kg)	143	0.45	0.07–1.26	0.17
BMI at T0 (kg/m^2^)	143	25.75	21.40–30.30	1.93
TFEQ‐disinhibition^c^	135	5.31	1.00–13.00	2.60
TFEQ‐restraint^c^	136	13.88	2.98–19.00	2.98
Food craving score^c^	137	0.53	0–1.57	0.36
Percent CR achieved^c^	135	19.48	−4.87–37.53	8.72
12‐week weight loss (kg)				
3 months vs. 0 months	141	6.44	−3.10–12.30	2.20
Subsequent weight loss after month 3 (kg)				
6 months vs. 3 months	137	1.69	−1.30–12.60	1.56
12 months vs. 3 months	130	2.82	−3.80–15.20	2.44
18 months vs. 3 months	121	2.77	−6.10–13.55	2.57
24 months vs. 3 months	119	2.17	−6.50–11.50	2.83

Abbreviations: CR, caloric restriction; HS, high school; *M*, mean; T0, initial time point; TFEQ, The three‐factor eating questionnaire; WV, weight variability.

^a^ Ethnicity was not used in the analyses due to low representation of minority groups.

^b^ Education was not used due to multicollinearity with income.

^c^ Time‐varying variables descriptive statistics are estimated at 6 months.

Additionally, WV was not significantly correlated with any other continuous independent variable (e.g., 12‐week weight loss, disinhibition, craving), whereas 12‐week weight loss was correlated with BMI, age, restraint, and percent CR achieved (Table 2).

**TABLE 2:** Between‐person correlations matrix of independent variables.

Variable	1	2	3	4	5	6	7	8
Standardized WV	—							
2 12‐week weight loss	−0.09	—						
3 BMI at T0	0.12	0.27**	—					
4 Age	−0.09	0.38**	0.15	—				
5 TFEQ‐disinhibition	0.15	−0.21	−0.08	−0.14	—			
6 TFEQ‐restraint	0.05	−0.10**	0.00	0.14	0.24**	—		
7 Food craving score	0.12	−0.14	0.10	0.00	0.23**	0.26**	—	
8 Percent CR achieved	−0.05	0.20*	−0.11	0.04	0.01	−0.05	−0.02	—

*Note*: Significance levels: *p < 0.05; **p < 0.01. Analyses included both time‐varying and time‐invariant independent variables. For time‐varying variables, person‐level mean values across time were used to estimate bivariate correlations with other variables.

Abbreviations: CR, caloric restriction; TFEQ, the three‐factor eating questionnaire; WV, weight variability.

### 3.2 WV and Subsequent Weight Loss in a Sample of Individuals Without Obesity

Model parameters are reported in Table 3. Subsequent weight loss after month 3 is defined as the difference between weight at month 3 and follow‐up weights. Positive values indicate weight loss relative to month 3, while negative values indicate weight regain. The final models included a fixed effect of time, WV, their interaction, relevant covariates, random intercept, and random slope for linear time. In model A, higher standardized WV was significantly associated with less weight loss at 6 months (*B* = −0.38, 95% CI [−0.66, −0.11], *β* = −0.16, *p* = 0.007), after including relevant demographic, weight‐ and RCT‐related covariates. Similarly, in model B, the association remained similar after inclusion of time‐varying percent CR achieved and eating behaviors (inhibition, restraint, food craving) (*B* = −0.38, 95% CI [−0.63, −0.13], *β* = −0.16, *p* = 0.003). The absolute difference in the estimated weight loss at 6 months between participants at the 25th and 75th percentiles of WV was 0.45 kg, with a corresponding relative difference of 19.2% (Table S1).

**TABLE 3:** Prospective longitudinal analysis examining correlates of weight loss – *N* = 143 (first wave).

Subsequent weight loss after month 3 – centered at 6 months (kg)								
Model for the mean	Model A				Model B			
	*B*	SE	*β*	*p*	*B*	SE	*β*	*p*
Time	0.02	0.01	0.007	0.179	0.05***	0.01	0.02	< 0.001
Standardized WV (12 weeks)	−0.38**	0.14	−0.16	0.007	−0.38**	0.13	−0.15	0.003
Standardized WV × time	0.01	0.01	0.006	0.319	0.01	0.01	0.004	0.429
12‐week weight loss	−0.21**	0.08	−0.19	0.007	−0.33***	0.07	−0.30	< 0.001
BMI at T0	0.33***	0.08	0.26	< 0.001	0.39***	0.07	0.31	< 0.001
Age	0.03	0.02	0.08	0.271	0.03	0.02	0.09	0.170
Site (Ref = Site 1)								
Site 2	0.49	0.34	0.20	0.153	0.32	0.32	0.12	0.316
Site 3	0.77*	0.38	0.32	0.046	0.59	0.34	0.24	0.086
Gender (Ref = Male)								
Female	−0.16	0.33	−0.07	0.631	−0.41	0.31	−0.16	0.186
Income (Ref = < $40,000)								
$40,000–$79,999	0.49	0.51	0.20	0.343	0.26	0.47	0.11	0.580
> $80,000	0.15	0.53	0.06	0.778	0.12	0.48	0.04	0.803
Percent CR achieved	—	—	—	—	0.08***	0.01	0.27	< 0.001
TFEQ‐disinhibition	—	—	—	—	−0.06	0.04	−0.07	0.184
TFEQ‐restraint	—	—	—	—	−0.02	0.03	−0.03	0.513
Food craving score	—	—	—	—	0.12	0.25	0.02	0.627
Intercept	−6.62**	2.10		0.002	−8.01***	1.94		< 0.001

Model for the variance	Variance (SE)	
Residual	1.35 (0.12)	1.43 (0.13)
Random intercept	1.49 (0.31)	0.81 (0.25)
Random slope		
Time	0.01 (0.002)	0.008 (0.002)
Model statistics		
Log likelihood	−974	−913
AIC/BIC	1981/2049	1884/1967

*Note*: Significance levels: *p < 0.05; **p < 0.01; ***p < 0.001. Analyses included five waves of data – weight at 6, 12, 18, and 24 months (*N* = 143). Comparison reference groups: site = site 1, gender = male, income = < $40,000.

Abbreviations: *B*, unstandardized coefficients; CR, caloric restriction; SE, standard error; TFEQ, the three‐factor eating questionnaire; WV, weight variability.

In both models, higher subsequent weight loss after month 3 was positively associated with BMI at T0 (*B* = 0.39, 95% CI [0.26, 0.53], *β* = 0.31, *p* < 0.001) and negatively associated with weight change over the measured WV period or the first 3 months (*B* = −0.33, 95% CI [−0.46, −0.19], *β* = −0.30, *p* < 0.001). After inclusion of the percent CR achieved over time, site differences were no longer significant, and percent CR achieved was positively associated with subsequent weight loss, as expected (*B* = 0.08, 95% CI [0.05, 0.11], *β* = 0.27, *p* < 0.001). No demographic or eating behavior covariates were significant in either model.

### 3.3 The Effect of WV Over Time on Subsequent Weight Loss

**FIGURE 2:**
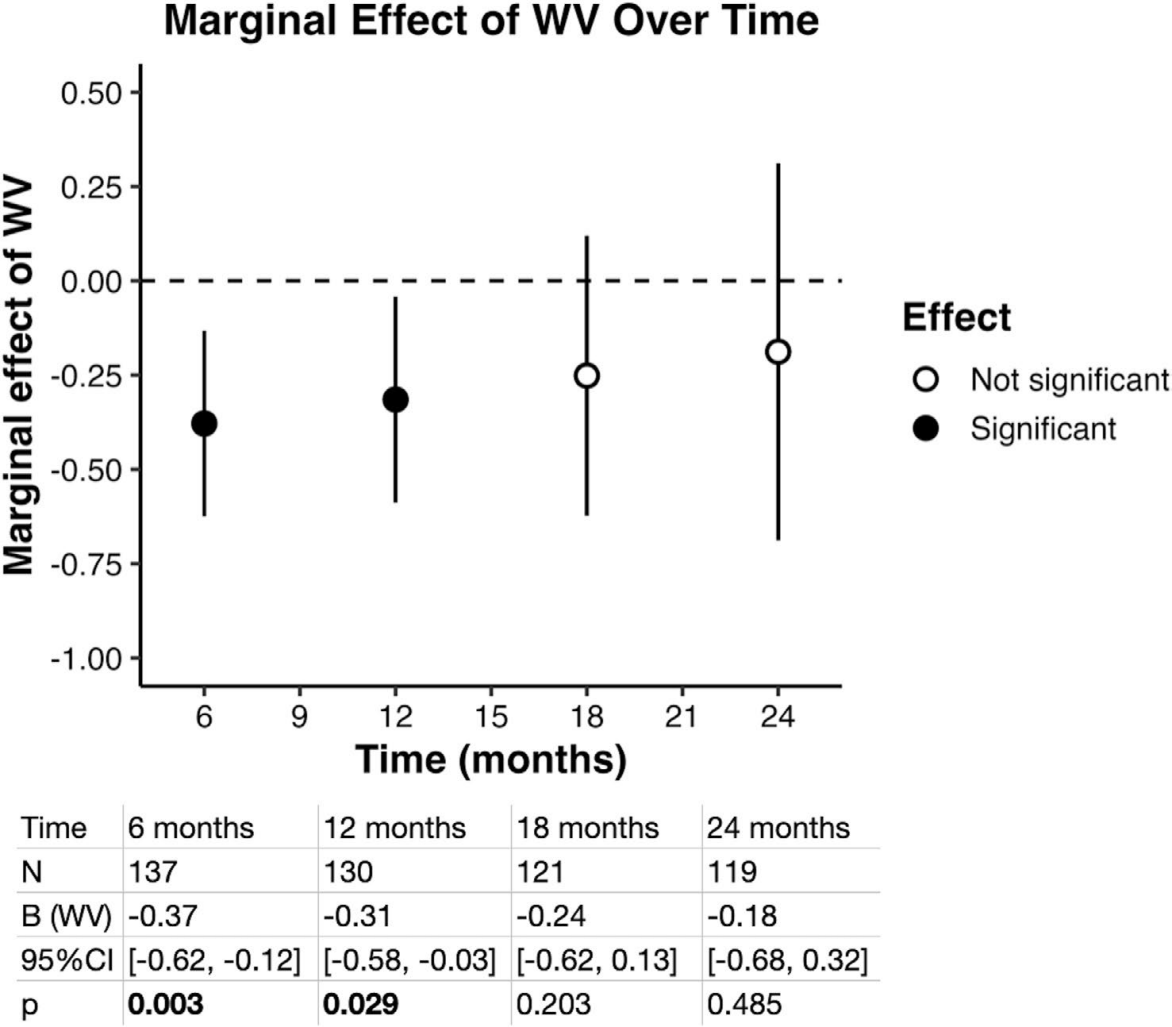
Marginal effects of WV over time.

The interaction between time and WV was not significant in either model (*B* = 0.01, 95% CI [−0.02, 0.04], *β* = 0.006, *p* = 0.429). Given limited power to detect this interaction, we also estimated marginal effects of WV over time. As shown in Figure 2 and Table S2, marginal effects remained negative throughout the study period but were no longer statistically significant after 1 year, as confidence intervals widened.

**FIGURE 3:**
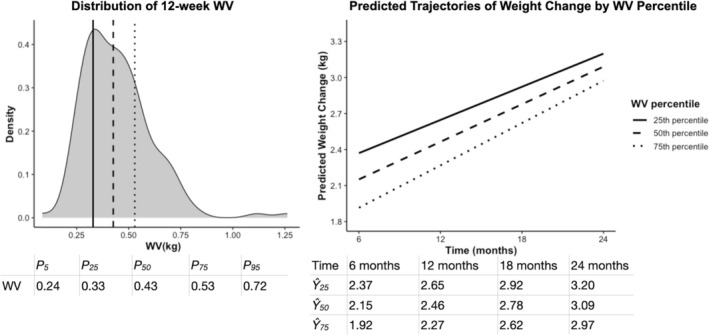
Distribution of 12‐week WV (left figure) and predicted weight loss trajectories over time (right figure).

Finally, to contextualize the effect of time and WV on the subsequent weight loss after month 3, we estimated predicted weight loss trajectories over time for participants at the 25th, 50th, and 75th percentiles of WV (Figure 3). Consistent with the previous results, the effect of WV weakens over time.

## 4 Discussion

### 4.1 Overview

In a sample of healthy adults without obesity who were on a prescribed 25% CR diet lasting 2 years as a part of the CALERIE study [23], higher WV was prospectively associated with subsequent lower weight loss at 6 months, after including weight‐related, demographic, study‐related, and eating behavior covariates. Secondary analyses provided tentative evidence that the effect persisted during the first year of the study, which subsequently appeared to weaken.

Prior literature has shown similar associations between WV and weight change among other populations, including in adults with elevated risk of weight gain [6], adults who were actively trying to lose weight either by themselves [9] or as part of the behavioral weight loss treatment [10], those who recently lost weight and were on the weight maintenance intervention [11], adolescents [8], and toddlers [7]. Diverse contexts and samples across which this association holds indicate that the WV effect is indeed robust and independent of health or obesity status.

Although the demonstrated effect of WV is small, it is important to study WV for at least two reasons. First, prior literature has demonstrated that WV is linked with not only weight change outcomes [4], but also with mortality and cardiovascular issues [12, 13, 15, 16, 17], metabolic syndrome [22], type 2 diabetes [18], and insulin resistance [20]. The effects of WV in these two independent literatures might be related, and if so, it is important to understand the full scope of WV's associations and synthesize knowledge across these disciplines. Second, WV might provide more insight into how the body achieves weight regulation around a stable body weight and help us to better understand how this seemingly random variation of WV has a persistent and robust predictive power across many health outcomes and populations.

### 4.2 Implications

We aimed to quantify the relationship between WV and subsequent weight loss in a not‐yet‐studied population of healthy adults without obesity undergoing weight loss. Taken together with research demonstrating an inverse association between WV and weight loss among individuals with obesity, our findings indicate that WV is prospectively associated with future weight change independent of obesity or health status. These results are best interpreted as predictive associations, whereas mechanistic interpretations and clinical applications remain speculative and require further investigation. The pervasiveness of WV's predictive power across highly diverse samples and conditions potentially suggests that WV is a unique, robust predictor of future weight change and as such may have potential clinical relevance. For example, individuals with a high standardized WV may be at higher risk for future weight gain or other adverse health outcomes, such as all‐cause mortality [14, 15, 16] or cardiovascular events [12, 13, 17]. Accordingly, these individuals could potentially be identified for closer monitoring or future preventive efforts.

WV could also have important theoretical utility by shedding light on the mechanisms of weight regulation. While the present findings demonstrate that WV predicts future weight change, interpretations regarding the underlying mechanisms remain speculative. The consistent and robust effects of WV on weight loss across settings and the developmental span are consistent with the hypothesis that the processes responsible for weight stability may occur automatically and outside conscious awareness. This is also supported by our replication and extension of the WV with future weight change relationship even after the inclusion of eating behaviors, percent CR achieved, and relevant covariates used or discussed before [4, 6, 7, 8, 9, 10, 25, 40]. Further, in addition to participants being unaware of their WV over 12 weeks, participants' goals were likely to lose weight and keep off all the weight they lost. The prediction of subsequent weight loss after month 3 by WV occurred in the context of these volitionally driven goals and in the absence of awareness of their status relative to others on the independent variable or on its relationship to their long‐term weight change.

In summary, the source of the WV effect remains a mystery, although it has been hypothesized that WV reflects a breakdown in homeostatic processes responsible for weight regulation [4, 44]. Investigating characteristics of this highly selective sample of adults without obesity or any notable physical or psychological conditions could further support that claim. This study ensured that our findings were not confounded by either a health condition or obesity status, suggesting that WV's association with weight change is robust across these factors and may extend across different populations. Importantly, the structured CR intervention and the exclusion criteria of the study (i.e., participation in high physical activity) likely reduced some sources of variability in energy intake and expenditure on weight and—therefore—WV. Similarly, the study design ensured that all weight‐related variables in our analyses were collected using standardized in‐clinic weighing protocols after fasting and voiding, so that short‐term variability due to food, fluid, bladder/bowel contents, time of day, and self‐report error is unlikely to explain these findings. Further, although we adjusted for percent CR achieved (i.e., a measure of adherence) and relevant eating behavior constructs, small day‐to‐day variations in fluid balance, unmeasured behaviors, physical activity, or other transient factors cannot be fully excluded. The present findings are, therefore, consistent with a conceptualization of WV as a biologically driven process [4, 44]. In further support of this argument, the WV effect on weight change over time is also observed among infants at ages 1 and 2 years old, who would have limited control over influences affecting their energy balance [7]. Accordingly, future studies should aim to elucidate the mechanisms underlying WV, including both biological processes and any residual behavioral contributions.

### 4.3 Future Directions

Several important questions remain for future research. First, the exact biobehavioral mechanisms that can explain WV's effect on weight change remain unknown. This might be an interdisciplinary effort that requires integration of biobehavioral and psychosocial factors in a model that can explain why some individuals have higher WV and, similarly, the relationship with adverse weight outcomes. Second, we need to standardize the methodology of calculating WV to understand how sampling frequency (e.g., daily, weekly, or monthly weights), integration window (i.e., duration of WV data collection), and prediction interval (e.g., 6 months vs. 2 years) affect the strength of the association between WV and future weight change. This, in turn, could reveal important practical trade‐offs for conducting research or for clinical applications in this field. Relatedly, although WV has been examined in individuals with bulimia nervosa [26] and anorexia nervosa [44, 45], this literature remains nascent and its implications for body image concerns and eating disorder treatment are unclear. It would also be beneficial to expand the list of outcomes that are potentially linked with WV, such as eating psychopathology, but also other psychological and physical health conditions. Finally, it is important to standardize WV methods across medical and weight change outcome disciplines to compare their findings once they all use the same unit of measurement (e.g., only short‐term or long‐term WV) and the same estimation strategy (e.g., RMSE vs. average successive variability). To summarize, these steps would allow us to confirm and measure the scope of WV's predictive power and to understand methodological considerations and mechanisms that explain these associations.

### 4.4 Strengths and Limitations

Our study was sufficiently powered to detect the small effect size of WV on future weight loss in the CALERIE sample. The scale and longitudinal nature of the design allowed us to examine patterns over time, controlling for important covariates. Additionally, we benefited from the CALERIE study being a well‐designed RCT that addressed threats to internal validity.

However, our study is not without limitations. This sample was relatively homogenous and resultingly, our findings might not generalize to non‐White, low‐income, or less educated adults. Establishing clear temporal ordering (no overlap between WV assessment period and subsequent weight loss relative to month 3) sacrificed information on total weight change (0–24 months). Additionally, the relatively small sample size limited power to test interactions between demographic factors and WV on future weight loss. Finally, we did not control for all behavioral (e.g., exercise) or eating‐related variables (e.g., food diary) that might have confounded our findings.

## 5 Conclusion

Here, we present evidence for the prospective and persistent association of WV and subsequent weight loss after month 3 in a sample of individuals without obesity who were part of the CALERIE study. These results replicate and extend prior findings in diverse populations, highlighting the robustness of WV as a predictor of long‐term weight change. WV may also serve as a unique marker for adverse psychological and physical health outcomes. Consequently, these findings raise important questions about the clinical and research implications, including how identifying individuals with higher WV might help to prevent negative health outcomes. More research is needed to understand the biobehavioral mechanisms underlying WV, which could provide insight into the resilience of humans' innate capacity for long‐term weight stability and inform potential clinical interventions.

## Author Contributions

**B.J.:** formal analysis, methodology, visualization, writing – original draft, writing – review and editing, investigation. **S.S.:** formal analysis, methodology, writing – original draft, writing – review and editing, **F.Z.:** methodology, writing – review and editing. **E.A.W.:** writing – review and editing. **S.B.R.:** data curation, writing – review and editing, funding acquisition, project administration. **M.R.L.:** conceptualization, investigation, writing – original draft, writing – review and editing, supervision.

## Funding

This project analyzed data from the CALERIE study that was collected as part of the National Institute of Aging grant R33AG070455.

## Conflicts of Interest

The authors declare no conflicts of interest.

## Supporting information


**Figure S1:** Normality assumption.
**Figure S2:** Homoscedasticity, linearity and independence of observation assumptions.
**Table S1:** Estimated weight loss at 6 months for participants at the 5th, 25th, 50th, 75th, and 95th percentiles of WV.
**Table S2:** Simple effect of WV at each follow‐up.

## Data Availability

Deidentified data from the CALERIE study is publicly available at https://calerie.duke.edu/. Use of this data was in accordance with the terms agreed upon the receipt. Our code is available by authors upon reasonable request.
