# Supplementary material for: Prospective Association Between Weight Variability and Subsequent Long‐Term Weight Loss in the CALERIE Study
Source: Obesity (Silver Spring). 2026 Jun 18;34(7):1409–19. doi: 10.1002/oby.70235 (PMC13306132; doi:10.1002/oby.70235)
Supplement: Supplementary file 1 — Figure S1: Normality assumption. Figure S2: Homoscedasticity, linearity and independence of observation assumptions. Table S1: Estimated weight loss at 6 months for participants at the 5th, 25th, 50th, 75th, and 95th percentiles of WV. Table S2: Simple effect of WV at each follow‐up. [file OBY-34-1409-s001.docx]

**Supplemental Material 1**

**Supporting Figures**

This supplemental material includes plots assessing the assumptions of the normality, linearity, homoscedasticity, and independence of observations; they were all met. Additionally, the degree of collinearity among the IVs was within an acceptable range (VIF < 5).

**Figure S1.** Normality assumption


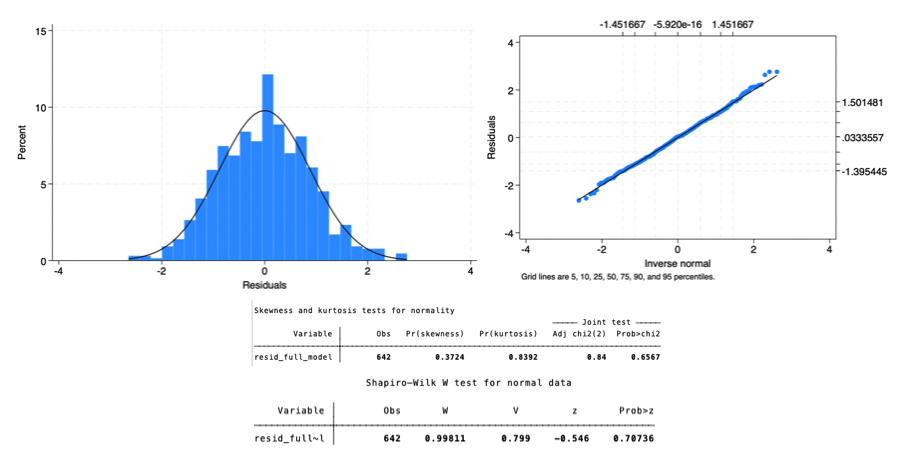


**Figure S2.** Homoscedasticity, linearity and independence of observation assumptions


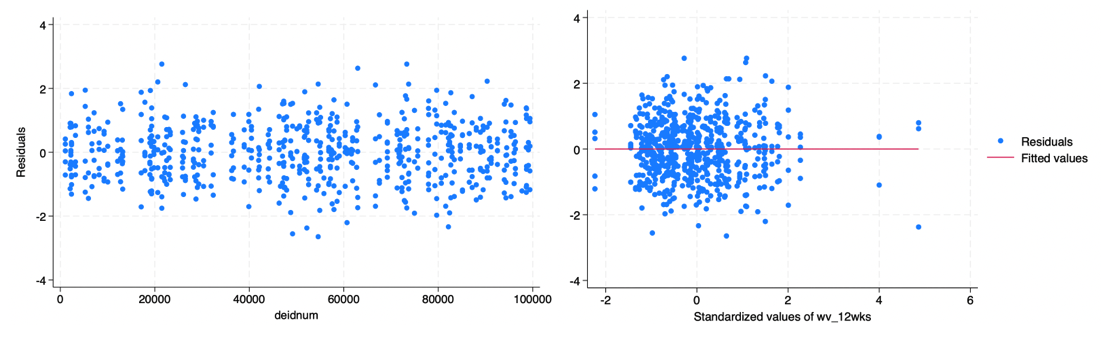


**Table S1.** Estimated weight loss at 6-months for participants at the 5^th^, 25^th^, 50^th^, 75^th^ and 95^th^ percentile of WV.

| WV Percentile | WV Value | Estimated weight loss at 6-months | Absolute difference (kg) | Percent difference (%) |
| --- | --- | --- | --- | --- |
| 5^th^ percentile | 0.24 | 2.56 | 0.19 | 8.0 |
| 25^th^ percentile | 0.33 | 2.37 | **Reference** | |
| 50^th^ percentile | 0.43 | 2.15 | -0.22 | -9.2 |
| 75^th^ percentile | 0.53 | 1.92 | -0.45 | -19.2 |
| 95^th^ percentile | 0.72 | 1.49 | -0.88 | -37.2 |

**Table S2.** Simple effect of WV at each follow-up.

| Time (months) | N | *B*(WV) | 95% CI | *p* |
| --- | --- | --- | --- | --- |
| 6 | 137 | -0.37 | [-0.62, -0.12] | 0.003 |
| 12 | 130 | -0.31 | [-0.58, -0.03] | 0.029 |
| 18 | 121 | -0.24 | [-0.62, 0.13] | 0.203 |
| 24 | 119 | -0.18 | [-0.68, 0.32] | 0.485 |

**WV Model Specification:**

Final 12-week WV model was:

*W_it_* = *𝛄_00_* + *𝛄_10_*Time_it_ + *𝛄_20_*Time_it_^2^ + *U_0i_* + *U_1i_*Time_it_ + *U_2i_*Time_it_^2^ + *ε_it_*

Where:



- *W_it_ –* weight of participant *i* at time *t* (in months) during the first 12 weeks.
- *𝛄_00,_ 𝛄_10,_ 𝛄_20_* – fixed effects (intercept, linear time, quadratic time)
- *U_0i_*_,_ *U_1i,_* *U_2i_* – participant specific random effects
- *ε_it_ ~ N*(0, σ^2^) *– residual error*

Residuals were obtained from a single mixed-effects model with a common fixed-effects structure and a participant-specific random intercepts and slopes. Weight variability (WV) for participant *i* was calculated as the root mean square error (RMSE) of participant-specific residuals from the 12-week mixed-effects model, where $n_{i}$ is the number of weight observations:

$${WV}_{i}=\sqrt{\frac{1}{n_{i}}\sum_{t=1}^{n_{i}} \varepsilon_{it}^{2}}$$

**WV R Code:**

# Optimal model after running anova command

m.6 <- lmer(weight ~ 1+wt_mo + I(wt_mo^2) + (1+wt_mo+I(wt_mo^2)|deidnum), data=wk12_wt_dataLONG, na.action="na.omit")

# Calculate residuals using the resid function.

residuals <- (resid(m.6))

res <- as.data.frame(residuals) # Convert the list of numbers to data frame format, the same as your original WV dataset

*wk12_wt_dataLONG <- cbind(wk12_wt_dataLONG, res) # Combine residuals - LONG format*

# Compute 12-week weight variability (WV) as RMSE of residuals per participant

RMSE12 <- ddply(wk12_wt_dataLONG,.(deidnum),summarize,

RMSE=sqrt(mean(residuals^2)))

summary(RMSE12$RMSE)

**Sensitivity Analyses for WV**

To evaluate the robustness of the RMSE-based WV method, which incorporates optimal fixed and random effects selected via likelihood ratio tests, we conducted sensitivity analyses using two alternative methods. First, we computed WV based on residuals from a linear-only detrending mixed-effects model, which included only linear fixed and random time effects, omitting the higher order terms. Participant-level variability was then calculated as the standard deviation of these residuals. While this simpler approach is easier to compute, it does not capture the nonlinear trajectories of weight change as effectively as the final RMSE-based quadratic method, which more accurately reflects deviations from each participant’s expected trajectory. Second, we computed average successive variability (ASV) from the residuals of the primary quadratic model, defined as the mean absolute difference between consecutive residuals for each participant. Both alternative WV metrics were strongly correlated with the primary RMSE-based WV, with the linear-only detrending WV showing a correlation of *r* = 0.77 and the ASV showing a correlation of *r* = 0.90. These results indicate that the RMSE-based WV is highly consistent with alternative approaches and theoretically the most appropriate measure for capturing participant-level weight fluctuations in this sample.
